# Physiological, Morphological, and Molecular Evaluation of Wheat Under Single (Drought, Salt, Heat) and Combined (Drought–Heat, Salt–Heat) Stress

**DOI:** 10.3390/ijms27115126

**Published:** 2026-06-05

**Authors:** Conghui Li, Xiaorui Guo, Lijuan Zhao, Enyang Mei, Yu Kang, Kangqi Xiang, Yuyue Zhang, Xueyu Lin, Xinmei Li, Shuqian Qian, Haitao Liu

**Affiliations:** 1 School of Biological Engineering, Huainan Normal University, Huainan 232038, China; zhaolijuan@hnnu.edu.cn (L.Z.); enyangmei@163.com (E.M.); ky951226@126.com (Y.K.); 17781419209@163.com (K.X.); 19556162344@163.com (Y.Z.); 13345626979@163.com (X.L.); lixinmei1218@163.com (X.L.); 18805827621@163.com (S.Q.); 2 Crop Genetics and Breeding Laboratory of Hebei, Institute of Cereal and Oil Crops, Hebei Academy of Agriculture and Forestry Sciences, Shijiazhuang 050031, China; guoxiaorui0404@163.com; 3 Key Laboratory of Bioresource and Environmental Biotechnology of Anhui Higher Education Institutes, Huainan Normal University, Huainan 232038, China

**Keywords:** wheat (*Triticum aestivum* L.), abiotic stress, combined stress, physiological response

## Abstract

Wheat (*Triticum aestivum* L.), a key grain food crop worldwide, faces increasing threats from combined abiotic stresses exacerbated by climate change. However, the comprehensive effects of drought, salinity, and high-temperature pressure on wheat seedlings remain poorly understood. Using the cultivar “Yannong 1212”, we conducted hydroponic experiments to investigate the physiological, morphological, antioxidant, osmoregulatory, membrane lipid peroxidation, and molecular responses of wheat seedlings to single and combined stresses, and then conducted multivariate statistical analyses. The results showed that drought or salt stress inhibited seed germination in a concentration-dependent manner. However, the combined stresses significantly inhibited germination and seedling growth, leading to leaf chlorosis, chlorophyll degradation, stomatal closure, and chloroplast damage. Physiologically, the combined effect of multiple stresses induced excessive ROS and MDA accumulation, promoted proline and soluble sugar synthesis, and triggered the dynamic responses of antioxidant enzymes. Drought stress increased SOD, POD, and CAT activities, whereas salt stress had the opposite effect, and combined stresses further increased SOD and POD activities, but reduced CAT activity. Additionally, stress-responsive genes were rapidly upregulated. Multivariate analyses confirmed that the combined stress of drought and heat was the most damaging. These findings explain the synergistic damage mechanisms of combined stresses, providing a theoretical basis for genetic improvement of wheat’s stress tolerance.

## 1. Introduction

In recent years, climate change, driven by global warming, has intensified abiotic stressors, including drought, salinity, high temperatures, and their combinations. These pressures severely damage plant growth, yield, and chloroplast development [1,2]. According to FAO data, over 40% of global wheat cultivation areas are exposed to periodic drought, causing an average yield reduction of 15–30% in affected years [3,4]. Soil salinization affects more than 800 million hectares of land worldwide, accounting for 20% of the total cultivated land area [5]. To defend against such harm, plants have evolved physiological and metabolic mechanisms, such as changing their root architecture, accumulating osmolytes, and boosting the activity of antioxidant enzymes to cope with single stresses [2]. However, multiple stresses pose a greater threat to agriculture than any single stress. Thus, understanding how multifactorial stress combinations affect plant growth is crucial for enhancing crop tolerance.

Multiple environmental stresses present a severe challenge to plant growth. Nevertheless, the responses of plants to combined stresses cannot be predicted from individual stress effects [6]. In natural environments, high temperatures often coexist with drought and salinity. Regions suffering from soil salinization are usually accompanied by water deficits, and drought, in turn, will further aggravate salt accumulation, exacerbating the adverse effects on crop growth [7]. Both stresses impose an osmotic imbalance on plant cells, trigger highly similar intracellular physiological disorders, and induce a sharp burst of reactive oxygen species (ROS) in plants [8,9]. Combined drought–heat and salt–heat stresses reduce stomatal conductance, photosynthesis, and chloroplast structure while increasing ROS and membrane damage. Among these combinations, heat often acts as the main stressor [10]. Plants experience more serious growth inhibition under combined stress, typically with additive effects [1]. Salt stress alone causes osmotic stress, ion imbalance, and oxidative damage, which decrease cellular function and crop yields [11]. For example, salt-sensitive rice mutant *shs1* has lower photosynthesis efficiency and accumulates much higher H_2_O_2_ levels under salt stress [12]. Additionally, combined salt and heat stress further suppresses photosynthesis by disrupting carbon assimilation and stomatal function [10,13]. Notably, in crops, including wheat, the combination of salt, heat, and drought leads to greater growth inhibition than single stresses [14].

Plants display distinct morphological responses to combined stress, including modifications to stomatal and chloroplast structure. Under drought or salt stress, plants usually close stomata to preserve water, whereas they keep them open to cool down [15]. However, under combined stress, they tend to close stomata to limit water transpiration, probably caused by ion imbalance and hyperosmotic effects [16]. Furthermore, multiple stresses trigger excessive ROS, which damages chloroplasts and mitochondria, leading to oxidative injury and impaired photosynthesis [17]. In addition, chloroplasts act as environmental sensors, producing ROS signals that trigger plastid-to-nucleus communication and assist plants in adapting to stress. Correspondingly, wheat evolves highly analogous antioxidant defense systems against oxidative stress under drought and salt treatments [18]. To counter oxidative damage, plants activate antioxidant enzymes, such as superoxide dismutase (SOD), catalase (CAT), and ascorbate peroxidase (APX), to maintain ROS homeostasis [19]. A variety of osmoprotectants are also synthesized and accumulated to stabilize cell structure and relieve osmotic stress [20]. However, combined stress can disrupt enzyme activity, leading to increased ROS generation, reduced membrane fluidity, and decreased photosynthetic capacity.

At the molecular level, plants adapt to adverse conditions by regulating gene expression [21]. Various genes and transcription factor families, such as *HSF*, *TaNAC2L*, *MYB*, and *WRKY*, support wheat tolerance to drought, heat, and salt [17,22,23]. In rice, the overexpression of *CspA* and *CspB*, which are RNA-binding bacterial chaperones, can improve stress tolerance [24]. In *Robinia pseudoacacia*, the expression level of Na^+^/H^+^ exchanger 1 (*NHX1*) and salt overly sensitive 1 (*SOS1*) was upregulated at low NaCl (50 mM), but declined at higher concentrations (100–200 mM) [25]. Additionally, salt and drought also rapidly activate the SnRK2 family of protein kinases in *Arabidopsis* [26]. The above findings demonstrate that each stress causes unique response mechanisms. But the molecular and physiological mechanisms underlying plant tolerance to combined stresses are still poorly understood.

Wheat (*Triticum aestivum* L.) is a globally important staple crop providing approximately 20% of the global dietary energy supply. According to the FAO, 790 million tons of wheat are produced annually, cultivated across about 210 million hectares worldwide [3]. Nevertheless, it is highly sensitive to heat, salt, and drought, especially at the emergence and seedling stages [27,28]. Therefore, improving stress resistance during these stages is vital for sustaining yields [29]. However, research into the combined effects of drought, salt, and heat on wheat is still limited. Therefore, in this study, we investigated the phenotypic, physiological, and molecular responses of wheat seedlings to single and combined stresses. Through these results, we aim to analyze tolerance mechanisms and provide a theoretical basis for understanding wheat’s response to multifactorial stresses.

## 2. Results

### 2.1. The Inhibition of Wheat Seed Germination and Growth Under Single and Combined Drought–Heat or Salt–Heat Stress

To determine the germination performance of wheat, seed germination was measured under single and combined stress treatments (Table 1). Drought stress (DS, using PEG for drought simulation) and salt stress (SS, using NaCl for salt simulation) inhibited germination in a concentration-dependent manner, with drought exerting a stronger effect. Under single drought stress, germination energy (GE) and germination percentage (GP) showed the most pronounced reductions at 20% PEG, decreasing by 15.22% and 10.84%, respectively, but reduced by 0.45% and 7.22% at 0.75% NaCl, respectively. Drought stress inhibited seed germination in a concentration-dependent manner. By contrast, single salt stress showed no obvious decreasing trend across the three concentrations in GE and germination index (GI), with slight fluctuations across NaCl concentrations. Notably, drought–heat combined stress (DHS) and salt–heat combined stress (SHS) induced far stronger inhibitory effects than single stress (*p* < 0.05). Compared with heat control (HS), GE, GP, and GI were reduced by 25.00%, 30.55%, and 19.86% at 20% DHS treatment and by 25.00%, 29.16%, and 17.19% under 0.75% SHS treatment. In summary, these results indicate that single drought stress inhibits wheat seed germination in a concentration-dependent manner. By contrast, single salt stress did not show a similar concentration-dependent inhibitory trend on GE and GI. Their combination with high temperature (DHS and SHS) exerted a synergistic inhibitory effect.

### 2.2. Morphology Analysis of Wheat Seedlings Under Single and Combined Drought–Heat or Salt–Heat Stress

**Figure 1:**
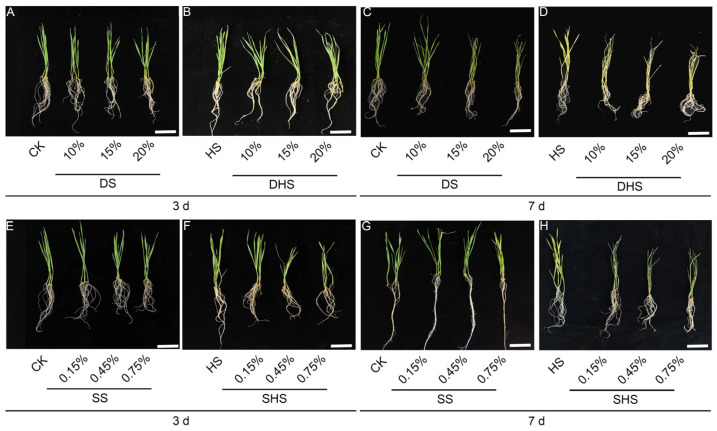
Changes in the phenotypes of wheat seedlings under single and combined stresses. (**A**,**B**), Changes in phenotypes under single DS and DHS stress treated for 3 days. (**C**,**D**), Changes in phenotypes under single DS and DHS stress treated for 7 days. (**E**,**F**), Changes in phenotypes under SS and SHS combined stress treated for 3 days. (**G**,**H**), Changes in phenotypes under SS and SHS combined stress treated for 7 days. CK: control group; DS: drought stress; SS: salt stress; HS: high temperature stress; DHS: drought stress with high temperature; SHS: salt stress with high temperature. Bars = 5 cm.

Wheat seedling growth was also evaluated under single and combined stress conditions (Figure 1 and Figure S1). Compared with the control, root length (RL) and leaf length (LL) decreased as PEG and NaCl concentrations increased, which was concentration-dependent (Table 2). After 7 days of single stress, RL and LL dropped by 22.67% and 10.79%, respectively, compared with the control (CK).

Notably, combined stress caused more severe inhibition of seedling growth. Under 20% DHS, LL and RL decreased by 18.34% and 26.62% after 3 days, and by 33.46% and 35.32% after 7 days, respectively. In contrast, 0.75% SHS caused reductions of 25.36% and 30.96% after 3 days, and 31.27% and 36.72% after 7 days, respectively (Table 2). Additionally, wheat seedlings under combined stress also displayed root desiccation, accompanied by leaf chlorosis and wilting (Figure 1E–H). In summary, these results show that combined stress synergistically aggravates physiological disorders, ultimately inhibiting shoot and root growth. Therefore, in subsequent analyses, we focused on severe drought–high temperature and salt–high temperature (20%PEG–high temperature for DHS and 0.75% NaCl–high temperature for SHS) combined stress treatments and corresponding single stress treatments (20%PEG for DS and 0.75% NaCl for SS) for further investigation.

### 2.3. Chlorophyll Content in Wheat Seedlings Under Single and Combined Stress

**Figure 2:**
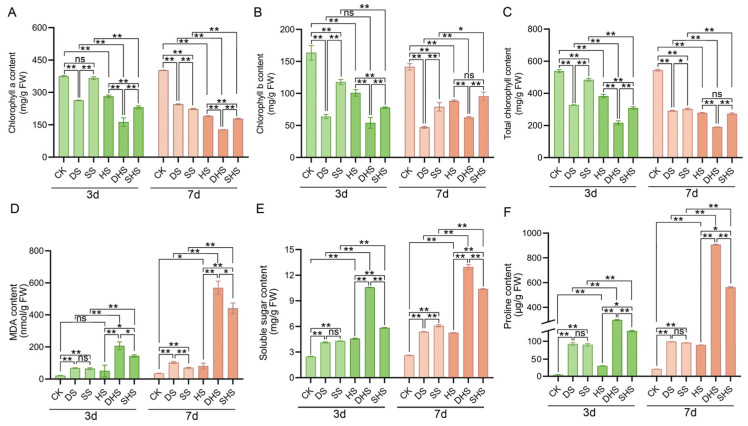
Variation in chlorophyll and osmotic adjustment substance contents of wheat seedlings under single and combined stresses. (**A**), Chlorophyll a, (**B**), chlorophyll b, and (**C**), total chlorophyll. (**D**), MDA content under single and combined stresses. (**E**), Soluble sugar content under single and combined stresses. (**F**), Proline content under single and combined stresses. All values are means ± SD (*n* = 3). Significant differences were determined using one-way ANOVA followed by Tukey’s HSD multiple comparison test and are indicated with asterisks as follows: * *p* < 0.05; ** *p* < 0.01; ns, no significant difference.

All stress treatments disrupted the balance between chlorophyll biosynthesis and degradation, altering chlorophyll content. To further investigate whether physiological activity changed underlying single and combined resistance in wheat, chlorophyll contents were tested (Figure 2). Under control conditions, chlorophyll a (Chl a), chlorophyll b (Chl b), and total chlorophyll all increased from day 3 to day 7, reflecting normal pigment accumulation (Figure 2A–C). In contrast, drought (DS), salt (SS), heat (HS), and their combined treatments (DHS/SHS) showed a significant decrease in Chl a, Chl b, and total chlorophyll relative to the control, with effects becoming more pronounced over time (Figure 2A–C). Compared with CK, DHS significantly reduced Chl a, b, and total chlorophyll by approximately 68.23%, 55.63%, and 64.89% after 7 days of treatment, respectively. Similarly, SHS reduced them by 55.83%, 32.39%, and 49.63%. In comparison, DS reduced these parameters by approximately 39.21%, 66.90%, and 46.32%, while SS reduced them by approximately 44.67%, 44.37%, and 44.49%, respectively. Notably, drought-related stresses (DS and DHS) exerted a greater inhibitory effect than salt-related stress, demonstrating a stronger impact on wheat seedling growth and physiological function. In conclusion, combined drought or salt stress with high temperature caused more severe damage to the photosynthetic pigment system, contributing to growth suppression.

### 2.4. Effects of Single and Combined Stresses on Physiological Indicators and Osmotic Regulatory Substances

Oxidative stress and osmotic regulation, induced by abiotic stress, reflect the degree of stress damage [30]. To analyze the physiological mechanisms underlying single and combined resistance in wheat, several physiological indicators were tested (Figure 2D–F). Malondialdehyde (MDA) content, a marker of membrane lipid peroxidation, was significantly higher under combined stress (DHS/SHS) than under single stress (Figure 2D). Compared with 7-day treated CK, MDA contents were elevated by 15.7-fold under DHS and 11.9-fold under SHS, respectively, which were higher than those in single DS and SS with 2.0 and 1.1-fold elevations, respectively.

Soluble sugars and proline, which are important osmoprotectants, exhibited similar accumulation patterns (Figure 2E,F). Compared with the control, all stress treatments significantly increased proline and soluble sugar content. However, individual drought and salt stress treatments had slight differences in soluble sugar content, whereas the DHS treatment resulted in a higher content than the SHS treatment (Figure 2E). Proline displayed similar accumulation patterns, with elevated contents under all stress treatments, but the highest levels were observed under combined stress, especially on day 7 (Figure 2F).

### 2.5. Effects of Single and Combined Stress on Antioxidant Activities of Wheat Seedlings

**Figure 3:**
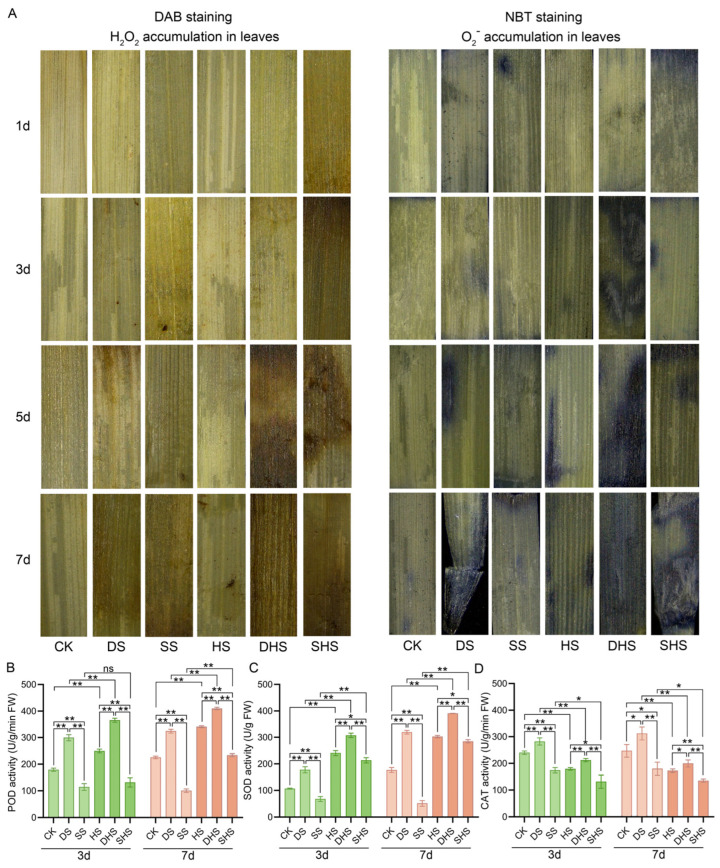
Detection of reactive oxygen species (ROS) in wheat seedlings under single and combined stresses. (**A**) H_2_O_2_ and O_2_^−^ accumulation in wheat leaves detected with DAB and NBT, respectively. (**B**–**D**) The activity of the antioxidant enzymes of POD (**B**), SOD (**C**), and CAT (**D**) under single and combined stresses. All values are given as means ± SD (*n* = 3). Significant differences were determined using one-way ANOVA, followed by Tukey’s HSD multiple comparison test and are indicated with asterisks as follows: * *p* < 0.05; ** *p* < 0.01; ns, no significant difference.

Abiotic stress can induce ROS production, including H_2_O_2_ and O_2_^−^. The accumulation of ROS was measured using 3,3′-diaminobenzidine (DAB) and nitro blue tetrazole (NBT) staining (Figure 3A, Figures S2 and S3). Regarding ROS accumulation, under salt and drought stress, H_2_O_2_ and O_2_^−^ contents were significantly higher than those in the control. DAB and NBT staining intensity increased with increasing stress concentration (Figures S2 and S3). Meanwhile, staining intensity increased with the extension of stress time, with the darkest staining observed under DHS and SHS treatments on day 7, indicating serious oxidative damage (Figure 3A). Notably, drought-related treatments (DS and DHS) induced more pronounced oxidative stress and osmotic adjustment responses than salt treatments, suggesting a stronger impact of drought stress on wheat seedlings.

Plants can mitigate stress-induced oxidative damage by enhancing antioxidant enzymes, including superoxide dismutase (SOD), peroxidase (POD), and catalase (CAT) [31]. To explore the synergistic relationship between antioxidant enzymes, the fresh leaves were used to measure their activities (Figure 3B–D). Under both single and combined stress conditions, drought treatments induced moderate increases in the activities of the antioxidant enzymes POD, SOD, and CAT. Compared with CK, POD, SOD, and CAT activities increased significantly by approximately 67.98%, 65.42%, and 17.99% at 3 days and 44%, 81.25% and 26.32% at 7 days, respectively. In contrast, single salt stress significantly decreased enzyme activities. Compared with the control, POD, SOD, and CAT activities were significantly decreased by approximately 31.47%, 5.96%, and 21.51%, respectively, after 7 days of treatment. Under combined stress conditions, POD and SOD activities increased by 0.82 and 1.22 times in drought and 3.56% and 61.36% in salt of POD and SOD activities, whereas CAT activity decreased by 19.43% and 45.34% under drought and salt at 7 days. These results indicated that antioxidant enzymes exhibit divergent regulatory mechanisms in response to different abiotic stresses. Drought stress effectively activates the antioxidant defense system by upregulating POD, SOD, and CAT activity. which, meanwhile, exhibited dynamic changes in response to single and combined treatments over the 7 days. Enzyme activities in most stress groups continued to rise from 3 to 7 days, with the most pronounced increases under DHS and SHS at 7 days. These findings collectively indicate that prolonged stress exposure further enhances the antioxidant defense response, with combined stress exerting a stronger stimulatory effect over time.

### 2.6. Effect of Single and Combined Stress on Stomata and Chloroplasts

**Figure 4:**
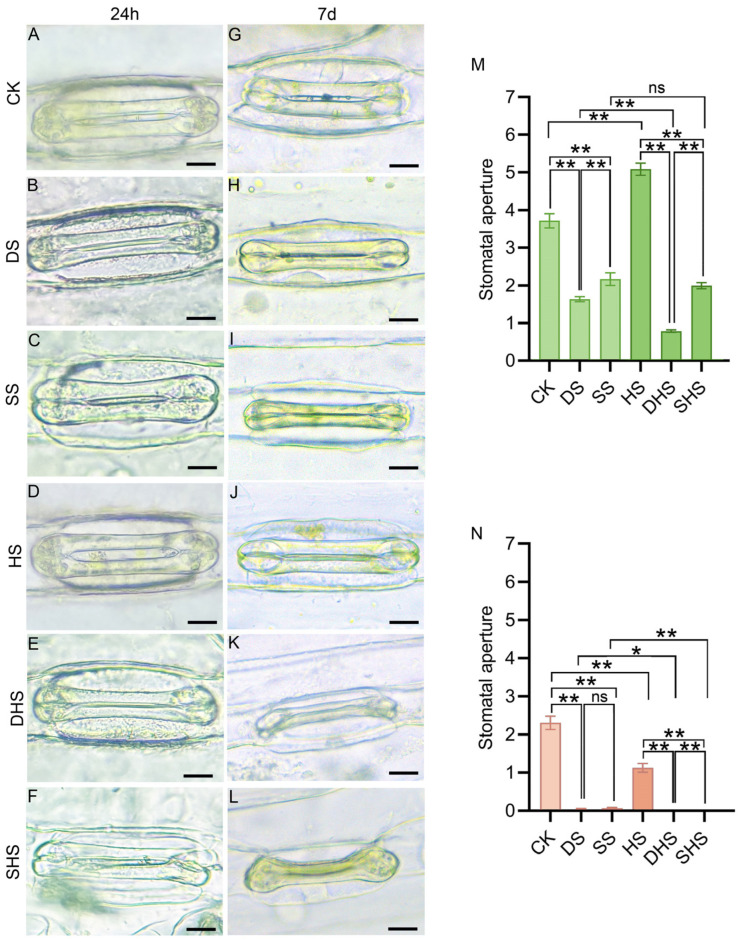
Effects on stomata of wheat under single and combined stresses. (**A**–**L**), Light microscopy image of stomata under different treatments. (**A**–**C**,**G**–**I**), Stromata in normal growth conditions. (**D**–**F**,**J**–**L**), Stromata in high-temperature conditions. (**M**,**N**), Quantitative analysis of stomatal aperture under different treatments at 24 h (**M**) and 7 d (**N**). CK: control group; DS: drought stress; SS: salt stress; HS: high temperature stress; DHS: drought stress with high temperature; SHS: salt stress with high temperature. Bars = 10 μm. All values are given as means ± SD (*n* = 3). Significant differences were determined using one-way ANOVA followed by Tukey’s HSD multiple comparison test and are indicated with asterisks as follows: * *p* < 0.05; ** *p* < 0.01; ns, no significant difference.

**Figure 5:**
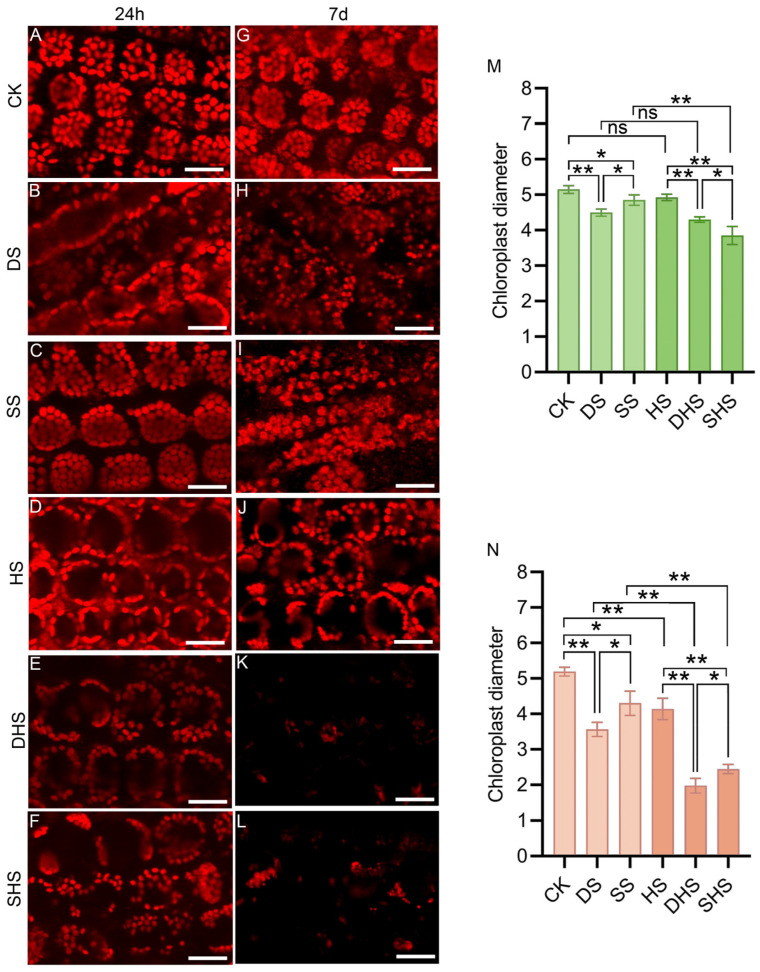
Effects on chloroplast morphology of wheat under single and combined stresses. (**A**–**L**), Light microscopy image of chloroplast morphology under different treatments. (**A**–**C**,**G**–**I**), Chloroplast in normal growth conditions. (**D**–**F**,**J**–**L**), Chloroplast in high-temperature conditions. (**M**,**N**), Quantitative analysis of chloroplast diameter under different treatments at 1 d (**M**) and 7 d (**N**). CK: control group; DS: drought stress; SS: salt stress; HS: high temperature stress; DHS: drought stress with high temperature; SHS: salt stress with high temperature. Bars = 20 μm. All values are given as means ± SD (*n* = 3). Significant differences were determined using one-way ANOVA followed by Tukey’s HSD multiple comparison test and are indicated with asterisks as follows: * *p* < 0.05; ** *p* < 0.01; ns, no significant difference.

Stomatal aperture and chloroplast size have important effects on plant growth and development [32]. Therefore, we observed the stomatal morphologies and chloroplast diameters under single and combined abiotic stress environments (Figure 4 and Figure 5). Drought and salt treatments caused moderate stomatal closure compared with CK at 1 day (Figure 4A–C), and most stomata closed at 7 days (Figure 4G–I). The opening of stomata was further exacerbated under combined stresses (DHS and SHS, Figure 4D–F,J–L).

Chloroplast red fluorescence imaging showed slight changes in chloroplast diameter after 24 h of stress, indicating intact chloroplast structure (Figure 5A–C). Compared with CK (5.1 μm), chloroplast diameter decreased to 4.5 nm under DS (12.6% reduction) and to 4.9 nm under SS (5.8% reduction) (Figure 5A–C,M). When combined with high temperature, the diameter decreased further to 4.3 μm (DHS) and 3.8 μm (SHS) compared with CK (5.1 μm) (Figure 5M). However, by 7 days, the red fluorescence intensity and size of chloroplasts diminished drastically (Figure 5G–I), especially under DHS and SHS, where signals became nearly undetectable, indicating severe chloroplast degradation and pigment loss (Figure 5J–L). Single DS and SS reduced diameters by approximately 31.4% and 17.2%, respectively, while DHS and SHS caused reductions of 62.0% and 31.4%, respectively (Figure 5N). These results collectively demonstrate that abiotic stresses, particularly in combination, induce significant stomatal closure and changes in chloroplast size, with prolonged exposure exacerbating chloroplast degradation and loss of photosynthetic capacity.

### 2.7. qRT-PCR Analysis of Stress-Responsive Gene Expression

**Figure 6:**
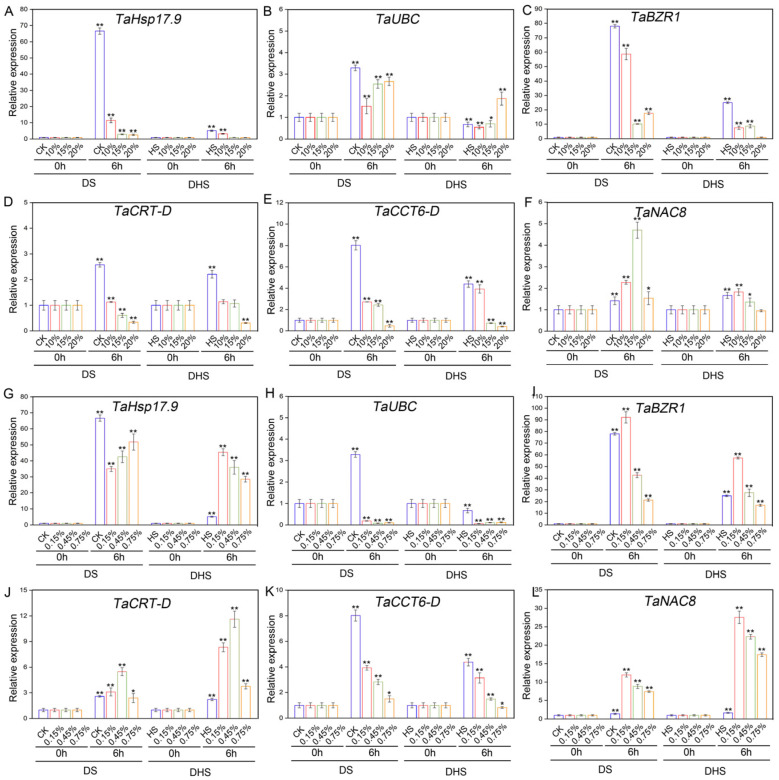
qRT-PCR analysis of the expression patterns of stress-responsive genes under single and combined stresses. (**A**–**F**), The expression patterns of *TaHsp17.9*, *TaUBC*, *TaBZR1*, *TaCRT-D*, *TaCCT6-D*, and *TaNAC8* under drought and drought–heat combined stress. (**G**–**L**), The expression patterns of *TaHsp17.9*, *TaUBC*, *TaBZR1*, *TaCRT-D*, *TaCCT6-D*, and *TaNAC8* under salt and salt–heat combined stress. Transcript levels were measured by qRT-PCR, and expression was normalized to the Actin gene. The relative expression level of each gene was calculated by using the 2^−∆∆Ct^ method. Relative gene expression was normalized to CK (0 h) for DS and SS groups and to HS (0 h) for DHS and SHS groups. All values are given as means ± SD (*n* = 3). Significant differences were determined using one-way ANOVA followed by Tukey’s HSD multiple comparison test and are indicated with asterisks as follows: * *p* < 0.05; ** *p* < 0.01.

Given the critical role of stress-responsive gene networks in plant adaptation to abiotic stress involving molecular chaperones, transcription factors, and signaling regulators [30], we performed qRT-PCR to detect their expression levels in wheat leaves under single and combined stress treatments at 0 h and 6 h (Figure 6). Under drought stress (DS-20%), *TaHsp17.9* and *TaBZR1* showed the most rapid and robust upregulation, with expression peaking at 2.6-fold and 17.6-fold higher than the control at 0 h. Under DHS, *TaCCT6-D* and *TaUBC* expression was further enhanced, especially *TaUBC*, which exhibited a significant increase under severe DHS (Figure 6B). In contrast, other genes exhibited more favorable responses under single stress and mild combined stress conditions. Under salt stress, *TaHsp17.9*, *TaBZR1*, *TaCRT-D*, *TaCCT6-D*, and *TaNAC8* were significantly upregulated at 6 h (Figure 6G–I). *TaHsp17.9* sustained upregulation under SS stress, with maximal induction of approximately 51.7-fold under 0.75% SS compared with the control at 0 h (Figure 6G). A similar expression pattern was observed under SHS treatment, where *TaHsp17.9* and *TaNAC8* showed strong responses to severe salt–heat stress (Figure 6A,F).

Furthermore, comparative analyses revealed that salt–heat stress generally elicited more rapid and intense transcriptional reactions for *TaHsp17.9*, *TaCRT-D*, and *TaNAC8*, whereas drought–heat stress brought about earlier activation of *TaUBC*. These results demonstrate that both single and combined stresses rapidly activated distinct groups of stress-responsive genes in wheat seedlings. The enhanced expression under combined stress suggests a synergistic regulatory network, indicating that the molecular pathways under drought and salt tolerance in wheat have shared genes.

### 2.8. Comprehensive Evaluation and Multivariate Analysis of Related Traits Under Single and Combined Stress Treatment

The membership function method was used to comprehensively evaluate wheat seedlings under single and combined stress, clarifying the core response indices and regulatory networks of wheat under stress (Tables S1 and S2). Germination indices, seedling morphology, and physiological parameters under different stress treatments were used as the basis for a comprehensive evaluation. The results showed that the membership values (MV) of the measurement index under DHS-20% and SHS-0.75% were significantly lower than those under other treatments, which were 0.227 and 0.328, respectively (Table S1). This result also indicated that combined treatment caused more severe damage to wheat seedlings.

**Figure 7:**
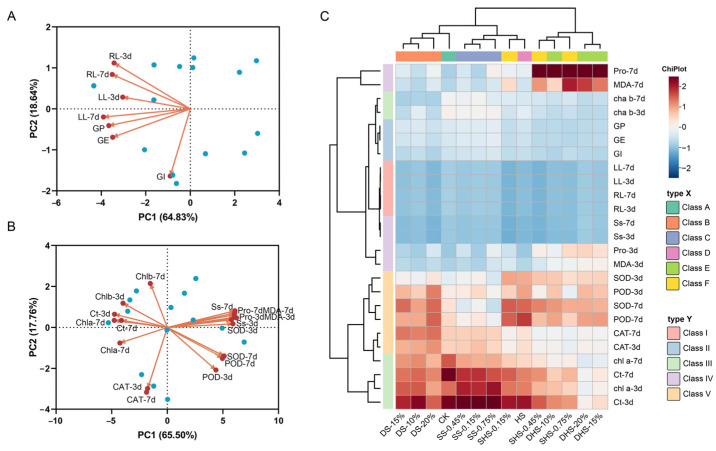
Multivariate analyses of wheat seedlings’ responses to single and combined stresses. (**A**,**B**) Principal component analysis index of (**A**) morphology and (**B**) physiological correlation. (**C**) Hierarchical clustering analysis of different stress treatments on wheat seedlings. Red color indicates higher values, and blue color indicates lower values. CK: control group; DS: drought stress; SS: salt stress; HS: high temperature stress; DHS: drought stress with high temperature; SHS: salt stress with high temperature. LL: leave length. RL: root length. GE: germination energy. GP: germination percentage. GI: germination index. Chl a: chlorophyll a content. Chl b: chlorophyll b content. Ct: total chlorophyll content. MDA: malondialdehyde. Pro: free proline. Ss: soluble sugar. POD: peroxidase activity. SOD: superoxide dismutase activity. CAT: catalase activity.

In this study, the morphological and physiological indicators of all treatment conditions were evaluated by principal component analysis (PCA) to reveal variation patterns among treatment groups (Figure 7A,B). The contribution rates of two principal components (PC1 and PC2) were 64.83% and 18.64% for morphological indicators, respectively, and 65.50% and 17.76% for physiological indicators, respectively (Figure 7A,B). For morphological indicators, RL-3d, RL-7d, and GE possessed the highest eigenvalues, significantly impacting both PC1 and PC2. GP and LL-7d had a large contribution to PC1, while GI had a high contribution to PC2 (Figure 7A). For physiological parameters, Chl b-3d and POD-3d were the main contributors. Pro, Ss, and Sp mainly contributed to PC2, with MDA having the least influence (Figure 7B). Total chlorophyll (Ct), MDA, Ss, Pro, and SOD-3d mainly contributed to PC1, with CAT and Chl b-7d greatly influencing PC2 (Figure 7B). Subsequently, a hierarchical cluster analysis was constructed to examine the relationships among morphological and physiological indices (Figure 7C). Hierarchical cluster analysis divided all treatments into six sub-clusters, clearly separating combined stress groups (DHS, SHS) from single stress and CK groups. Combined stress resulted in higher Pro, SOD, POD, and MDA levels after 7 days of treatment. Compared with SS treatment, Pro, SOD, POD, and MDA contents were also significantly increased under SHS stress after 7 days. Under single stress, CAT activity, chlorophyll a content, and total chlorophyll content were elevated, with the lowest levels observed under 20–DS. Growth indices (GP, GE, GI, LL, RL, Ss) exhibited a consistent trend, whereas high-concentration combined stress exerted an inhibitory effect on these indices. These results indicated that combined stress suppressed plant germination, growth, and development.

**Figure 8:**
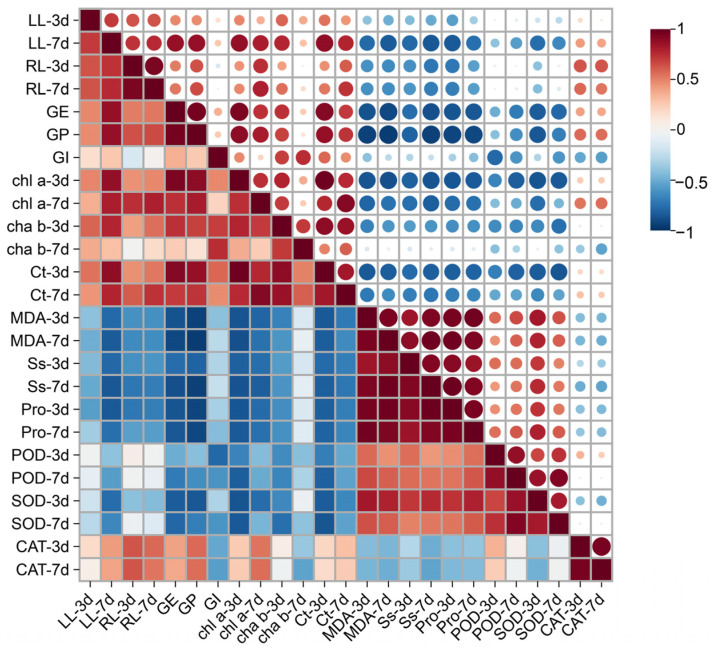
Correlation analysis of different stress treatments on wheat seedlings. Blue color indicates positive correlation, red color indicates negative correlation, and circular area indicates the magnitude of the correlation coefficient. LL: leave length. RL: root length. GE: germination energy. GP: germination percentage. GI: germination index. Chl a: chlorophyll a content. Chl b: chlorophyll b content. Ct: total chlorophyll content. MDA: malondialdehyde. Pro: free proline. Ss: soluble sugar. POD: peroxidase activity. SOD: superoxide dismutase activity. CAT: catalase activity.

Correlation analysis performed using a correlation matrix (Table S3) revealed the relationships between different indices in different stress treatments (Figure 8). Seed germination showed significant negative correlations with antioxidant enzyme activities (SOD, POD, and CAT) and the markers of oxidative stress (MDA, Pro, and Ss). Growth indexes (LL and RL) also had significantly negative correlation relations with oxidative stress markers (MDA, Pro, and Ss) and the activities of two antioxidant enzymes (SOD, POD), while positively correlating with CAT activity. In addition, among all morphological and physiological parameters, MDA-7d was strongly positively correlated with soluble sugar (r = 0.97) and proline (r = 0.94). The correlation between SOD and POD (but not CAT) was also extremely remarkable (r = 0.93). These results indicate that CAT has a specific protection function in seed germination and seedling growth under abiotic stress. In contrast, elevated SOD or POD activities and oxidative stress markers reflect stress-induced damage that negatively regulates germination and growth. Furthermore, chlorophyll content showed a positive correlation with LL, RL, and seed germination, while it displayed a negative correlation with SOD and POD activities, indicating that seedling growth was closely connected with photosynthetic capacity. Strong correlations also existed between MDA and SOD, MDA and POD, SOD and Ss, and CAT and RL, while the connections between CAT, GI, Ss, Pro, and MDA were comparatively weaker (Figure 8). These findings confirm that wheat seedlings respond to abiotic pressure via a cooperative network involving lipid peroxidation, osmotic adjustment, and the antioxidant enzyme system. In the meantime, CAT acted as a relatively independent regulator that promotes germination and growth.

## 3. Discussion

### 3.1. Seed Germination and Morphology of Wheat in Response to Single and Combined Stresses

In the wheat life cycle, the germination stage is the most stress-sensitive to external environmental conditions [33]. In the present environmental context, plants are frequently exposed to multiple abiotic stresses and their combinations [6,34]. Drought, salt, and high temperature markedly impair seed germination and metabolism through diverse mechanisms [35]. High temperature stress has been shown to significantly inhibit seed germination in chickpea, rice, and beans [11,36,37].

In this study, single drought and salt stresses inhibited wheat seed germination in a concentration-dependent manner (Table 1), which was consistent with Wang et al. [11]. Notably, combined drought–heat (DHS) and salt–heat (SHS) stresses induced synergistic inhibitory effects. For example, 20–DHS reduced GP, GE, and GI to 55.56%, 56.67%, and 51.75%, respectively, impacts far exceeding those of single stresses (Table 1). Additionally, combined stress also reduced plant height and root length more severely than single stresses, along with severe phenotypic damage, including root desiccation and leaf chlorosis/wilting (Figure 1 and Figure S1). Sachdev supports this synergism [30], highlighting that combined stresses exacerbate physiological damage by disrupting osmotic balance and accelerating ROS-mediated lipid peroxidation, with high temperature acting as a critical stress amplifier that exacerbates physiological damage beyond the sum of single stress effects. This is consistent with field observations that salinity and drought are often accompanied by heat stress, leading to chronic stress that reduces productivity through affecting osmotic imbalance, ion toxicity, and cell growth [38].

### 3.2. Physiological and Biochemical Response of Wheat to Single and Combined Stresses

Abiotic stress induces excessive ROS production, leading to membrane lipid peroxidation and ultimately limiting plant growth [32]. The result showed that both single and combined stresses significantly increased ROS, including H_2_O_2_ and O_2_^-^ accumulation and MDA content in wheat; hence, combined stresses (especially drought–heat) brought more serious damage (Figure 2D, Figure 3A, Figures S2 and S3). These results are consistent with previous reports that prolonged stress disrupts the balance between ROS generation and removal, leading to membrane damage, and inhibits antioxidant enzyme activity [39]. Under adverse environments, plant cells rapidly accumulate osmolytes, such as proline and soluble sugars, to maintain water balance [40]. Our study observed that higher osmotic accumulation appeared under combined stresses than under single stresses (Figure 2E,F), consistent with reports that combined salt, drought, and heat stress significantly increases proline and soluble sugar contents [6,41], indicating that osmoprotectant accumulation is closely correlated with stress intensity.

Plants regulate antioxidant enzymes (SOD, CAT, POD) to balance ROS production and scavenging to fight oxidative damage [42]. In our study, antioxidant enzyme activities were significantly upregulated initially under both single and combined stresses, along with increased MDA content (Figure 3B–D). However, in the combined stresses of heat–salt and heat–drought, their activities remained increased in DHS. Nevertheless, they were higher under HS than under SS or SHS, indicating that high temperature and combined stress can disrupt the synergistic effect of the antioxidant enzyme system, ultimately leading to oxidative stress. These results reflect the dynamic balance between ROS production and scavenging in wheat seedlings [43], but severe combined stress exceeds the scavenging capacity of the antioxidant system, leading to ROS accumulation and increased lipid peroxidation [6]. Our results consistently show that DHS, especially 20% DHS stress, induced stronger adverse impacts on wheat growth, physiology, and gene expression than SHS. Drought reduces soil water availability and restricts root water uptake, imposing a rapid and severe osmotic shock, while salt stress involves a more gradual osmotic component followed by ionic toxicity. Meanwhile, high temperature further accelerates intracellular water loss and leaf transpiration, disrupts antioxidant enzyme activity, and exacerbates ROS bursts triggered by drought, leading to more serious oxidative damage and metabolic disorder [9], making DHS the most harmful stress combination for wheat seedlings. Furthermore, the qRT-PCR results showed that genes involved in antioxidant defense and stress signaling were clearly more upregulated under combined stresses than single stresses (Figure 6), consistent with the physiological and biochemical alterations observed, confirming the coordinated regulation of functional genes and physiological metabolism [41].

### 3.3. Stomata and Chloroplast Responses to Single and Combined Stresses of Wheat

Stomata are the main regulators of transpiration and water balance, while chloroplasts function as the core photosynthetic machinery, which are highly sensitive to abiotic stress [41]. The results showed that single stresses (drought, salt, heat) all caused significant damage to stomata and chloroplasts, with varying severity (Figure 4 and Figure 5). Single drought and salt stress induced stomatal closure to maintain cellular water balance, with more obvious closure under salt stress, while heat stress displayed inconsistent stomatal responses, likely due to differences in genotypic or experimental conditions (Figure 4) [44]. Simultaneously, drought stress induced chloroplast deformation and diameter reduction (Figure 5), consistent with the findings of Suliman, who discovered that drought damages chloroplast integrity and disrupts photosynthesis [44].

Notably, combined stresses caused significantly more severe damage to stomata and chloroplasts than single stresses, a conserved response across plant species that reflects molecular regulation of combined stress signals (Figure 4 and Figure 5) and involves intricate molecular regulatory networks [45]. Similar stomatal responses have been observed in *Arabidopsis*, tomato, broadleaf evergreens, and soybean, suggesting evolutionary conservation [6,46,47,48]. In addition, combined salt–heat, drought–heat, or triple stresses further aggravated chloroplast damage and chlorophyll degradation (Figure 2 and Figure 5) [13,49], consistent with the decline in Chl a, Chl b, and total chlorophyll (Figure 2). Structural damage and substantial chlorophyll reduction under combined stresses are the key causes of growth inhibition. Similar results were also recorded in tomato, maize, and Solanum under combined stress conditions [13,49,50].

### 3.4. Differential Gene Regulation Modulates Wheat Tolerance to Diverse Abiotic Stress Combinations

In this study, we detected changes in the expression of six stress-related genes by qRT-PCR (Figure 6). The results revealed distinct expression patterns of six stress-related genes (*TaHsp17.9*, *TaUBC*, *TaBZR1*, *TaCRT-D*, *TaCCT6-D* and *TaNAC8*) under single and combined stresses, which are closely linked to the morphological and physiological variations. *TaHsp17.9*, *TaUBC*, *TaBZR1*, and *TaNAC8* showed increased expression under both single-drought- and salt-treated conditions, consistent with increased antioxidant enzyme activity and osmoprotectant contents to alleviate oxidative damage [51]. In contrast, among the six genes, only *TaUBC* was induced by combined drought–heat stresses; the others were downregulated or showed no significant difference. In addition, all six genes except *TaUBC* were strongly upregulated under single salt stress and combined salt–heat stress. This contrasting expression trend suggests that combined stress may disturb normal cellular metabolism and stress signaling, aggravating growth inhibition and impairing physiological homeostasis. This phenomenon has also been observed in previous studies on cereal crops exposed to multiple abiotic stresses [52]. The differences between drought–heat and salt–heat stress further illustrate the complexity of plant stress response mechanisms.

### 3.5. Multivariate Comprehensive Evaluation of Combined Stress on Wheat Seedlings

Plant tolerance to single and combined stress is complex and multifaceted [53]. To comprehensively evaluate stress effects on physiological and biochemical responses, we performed multi-dimensional statistical analyses. The MV method, which is extensively utilized to examine stress tolerance, allowed standardization of germination, morphological, and physiological–biochemical indices, enabling the measurement of plant stress tolerance [27,54]. Notably, DS and DHS displayed lower MV values than SS and SHS (Table S1), suggesting wheat seedlings are more sensitive to drought and heat stress than to salt stress, consistent with the studies on oat and cotton [53,55].

Similar to the significant correlation between biochemical and physiological traits reported in mung bean by Alsamadany [1], our study confirmed this correlation in wheat seedlings under different stress treatments (Figure 7 and Figure 8). The PCA analysis showed that root length and germination energy were the most influential morphological indicators. At the same time, chlorophyll content and POD and SOD activities were the core physiological factors driving the variation in stress response, highlighting these indicators as pivotal for stress adaptation (Figure 7A,B and Table S2). Consistent with our results, the synergistic network of POD, CAT, SOD, and APX acts as a core system for antioxidant defense, with its activation positively linked to seed germination and seedling growth [31]. Correlation analysis further unraveled the internal regulatory network of wheat seedling stress responses (Figure 8 and Table S3). MDA showed a strong positive correlation with osmoprotectants, and SOD with POD activity. Seed germination and seedling growth traits (LL and RL) were obviously negatively correlated with oxidative stress markers and SOD/POD activity, but positively correlated with CAT activity. These findings indicate that wheat seedlings adapt to abiotic stress via a coordinated network of lipid peroxidation, osmotic adjustment, and the SOD-POD system, while CAT plays a unique protective function in keeping germination and growth, clarifying the distinct regulatory functions of antioxidant enzymes in stress tolerance. Simultaneously, the hierarchical clustering separated the combined stress groups from the single stress and control groups, verifying the specific synergistic effects of drought–heat and salt–heat stresses (Figure 7C). These results are consistent with previous studies and indicate significant interactive effects in the morphological indicators and physiological–biochemical parameters [1,14,29].

**Figure 9:**
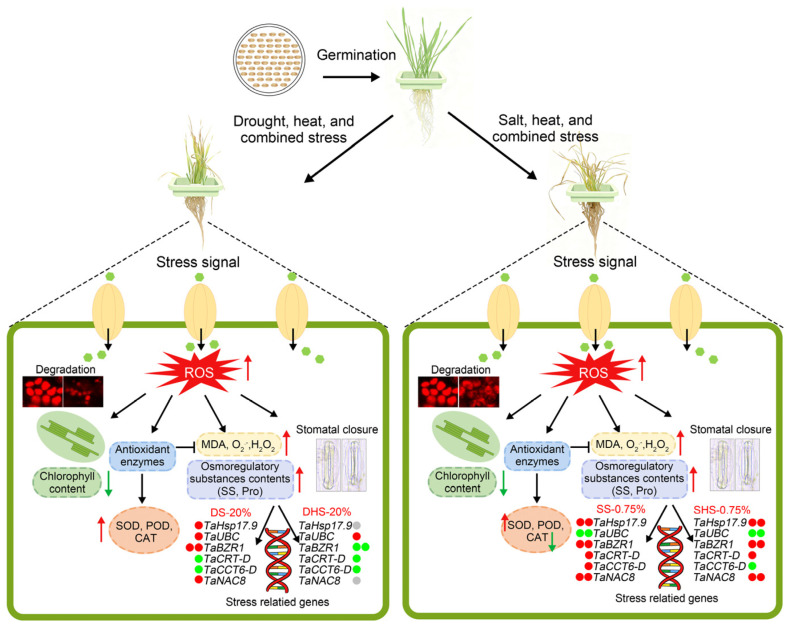
Schematic model of plant responses to single and combined drought–heat and salt–heat stresses. (**Left**): drought, heat, and combined stress. (**Right**): salt, heat, and combined stress. Stress signals trigger excessive ROS accumulation, leading to chlorophyll decline, stomatal closure, and increased MDA, O_2_^−^, and H_2_O_2_ in both drought–heat and salt–heat combined stresses. Osmoregulatory substances accumulate to maintain cellular homeostasis. Antioxidant enzymes (SOD, POD, CAT) and stress-related genes are differentially regulated. This reveals distinct adaptive mechanisms under drought–heat vs. salt–heat stresses. Red upward arrows indicate significant increases, and green downward arrows indicate significant decreases in physiological parameters under stress compared with the control. Gene expression patterns of stress-related genes are labeled with color-coded boxes: red for upregulated, green for downregulated, and grey for no significant change. The number of color-coded boxes indicates the intensity of gene expression changes.

Collectively, combined stress exhibited lower growth parameters and higher MDA, H_2_O_2_, and O_2_^–^ levels, suggesting that combined stress exerted synergistic inhibitory effects on wheat seedlings through breaking osmotic balance, inducing serious oxidative damage, damaging chloroplast structure, and altering expression of stress-related genes. Specifically, excessive ROS accumulation and impaired antioxidant defense triggered a series of physiological injuries. These processes further resulted in stomatal closure and chloroplast damage and a significant reduction in photosynthesis, ultimately inhibiting plant growth and development (Figure 9).

Taken together, these findings also highlight important practical relevance for wheat stress-tolerance breeding. The distinct physiological and molecular response characteristics of wheat under single and combined stresses revealed in this study provide indicators for germplasm screening. The stress-responsive genes identified here represent potential targets for gene editing. However, several limitations should be acknowledged. All experiments were carried out under controlled laboratory conditions and focused solely on the seedling stage of wheat, lacking field environment data and reproductive stage observations. Further investigations should be performed using multiple wheat types, incorporate field validation, and investigate the performance of stress-tolerant wheat materials at the reproductive stage. Moreover, time-series transcriptomics and metabolomics will be valuable for illuminating the molecular regulatory networks of wheat’s response to multiple abiotic stresses.

## 4. Materials and Methods

### 4.1. Plant Materials and Growth Conditions

The wheat variety “Yannong 1212” was selected for this experiment. Uniform-sized wheat seeds were surface-sterilized with a 75% alcohol solution for 1 min, then rinsed with ultrapure water 5 times. The seeds were then placed in Petri dishes and transferred to an artificial climate chamber (PGX-450C, Ningbo, China) at 26/20 °C under a 16 h/8 h (day/night) cycle, 60% relative humidity and a photosynthetic photon flux density (PPFD) of 120 µmol m^−2^ s^−1^. After the seeds sprouted, they were individually planted in seedling pots and transferred to an artificial climate chamber for acclimation for 1 week. Then, the uniform-sized wheat seedlings were divided into sixteen groups of 30 seedings per pots and treated in a completely randomized design as follows: (1) control (CK): water; (2) drought stress (DS): irrigated with 10%, 15%, and 20% PEG-6000 solution per pot; (3) salt stress (SS): irrigated with 0.15%, 0.45%, and 0.75% NaCl solution per pot; (4) heat stress (HS): control plants grown at 37/30 °C (day/night); (5) combined drought and heat stress (DHS): irrigated with 10%, 15%, and 20% PEG-6000 solution per pot and grown at 37/30 °C (day/night); and (6) combined salt and heat stress (SHS): irrigated with 0.15%, 0.45%, and 0.75% NaCl solution per pot and grown at 37/30 °C (day/night). After 3 and 7 days of stress treatment, morphological parameters were measured, and the plant leaf samples were collected for the follow-up experiments. The experiment was repeated three times.

### 4.2. Analysis of Seed Germination of Wheat

Wheat seedlings were sterilized, washed, and cultured in water (as a control) or different concentrations of PEG-6000 and NaCl. The seeds were then germinated and grown on filter paper under standard growth conditions or under combined high-temperature stress for 7 days. The seeds were germinated in a growth chamber (26/20 °C or 37/30 °C day/night temperature cycle, 16/8 h light/dark cycle with light intensity of 120 umol m^−2^ s^−1^) for the indicated periods of time. Seed germination potential, rate, and index were calculated, and photographs were taken at 3 and 7 days after cultivation [5].

### 4.3. Measurement of Physiological and Biochemical Indicators

Chlorophyll content was measured via the UV-absorption method. Samples were extracted by adding 5 mL of buffered alcohol (95% alcohol) to 1 g of fresh leaf weight and centrifuged for 10 min at 15,000× *g* after incubation overnight at 4 °C. The concentration of chlorophyll in the samples was determined as previously described and expressed according to the method of Porra et al. [56]. The method described by Sarker was used to estimate soluble sugar (Ss) content. Briefly, 0.5 g of the leaf sample was added to 9 mL of distilled water in a boiling water bath for 40 min [57]. A total of 1 mL of the supernatant was mixed with 5 mL of sulfuric acid-anthrone reagent, then boiled for 10 min and cooled. The absorption value was measured in a spectrophotometer at 625 nm. Free proline (Pro) content was determined as described by Li using the acid ninhydrin assay [58]. MDA content was measured using the thiobarbituric acid method [59].

Superoxide dismutase (SOD) and peroxidase (POD) activities were visualized via nitro blue tetrazolium (NBT) and guaiacol colorimetry, respectively [60]. Catalase (CAT) activity was quantified via the ultraviolet absorption method [60]. About 0.5 g of fresh wheat leaves was weighed and then ground in a pH 7.8 sodium phosphate buffer (PBS). The homogenate was centrifuged at 4 °C for 10 min at 10,000 rpm to obtain the crude enzyme extract, with three replicates per treatment.

### 4.4. Observation of Chloroplast and Stomatal Morphology

Wheat seedlings under different treatments were cultivated and collected at 3 and 7 days, and then cut into segments approximately 1 cm in length. These tissues were then fixed in 3.5% glutaraldehyde solution for 24 h in darkness. Subsequently, the samples were immersed in 0.1 mol/L Na_2_-EDTA solution (pH 9.0) and incubated in a water bath at 60 °C for 2 h. All sections were examined by electron microscopy (Nikon, Tokyo, Japan). Images of chlorophyll autofluorescence under different treatments were obtained by confocal laser scanning microscopy (FV3000, Olympus, Tokyo, Japan). Chlorophyll autofluorescence was visualized using 633 nm excitation with a 650–750 nm laser. The chlorophyll autofluorescence diameter was measured using ImageJ software (v1.54f, https://imagej.net/ij/).

### 4.5. qRT-PCR

Total RNA was isolated from wheat leaves subjected to different treatments using the FastPure^®^ Plant Total RNA Isolation Kit (Vazyme, Nanjing, China) according to the manufacturer’s instructions. First-strand cDNA was synthesized using the TRUEscript RT Kit (+gDNA Eraser) (Aidlab Bio, Beijing, China). Quantitative reverse transcription PCR (qRT-PCR) was performed using the Taq Pro Universal SYBR qPCR Master Mix (Vazyme Biotech, Nanjing, China) following the manufacturer’s protocol on a Step One Plus Real-time PCR System (Applied Biosystems, Foster City, CA, USA). To analyze the gene expression levels under single stress (salt or drought) and combined stress (salt–high temperature or drought–high temperature), six stress-responsive genes were selected (*TaHsp17.9*, *TaUBC*, *TaBZR1*, *TaCRT-D*, *TaCCT6-D*, and *TaNAC8*). Sequence data can be found in the NCBI database under accession numbers MN684335 (*TaHsp17.9*), AY736121 (*TaUBC*), KAF7000297 (*TaBZR1*), EF452301 (*TaCRT-D*), KAF7017159 (*TaCCT6-D*), and KAF7006672 (*TaNAC8*). Gene-specific primers for these six genes are shown in Supplementary Table S4, and the *actin* gene was used as the internal control for normalization. Relative expression level of each gene was calculated using the 2^−ΔΔCt^ method. The relative gene expression was normalized to CK (0 h) for the DS and SS groups and to HS (0 h) for the DHS and SHS groups. The 2^−∆∆Ct^ method of calculation was:

Ratio (test/calibrator) = 2^−[Ct(target, test) − Ct(ref, test)] − [Ct(target, control) − Ct(ref, control)]^

### 4.6. Comprehensive Evaluation and Statistical Analysis

Each treatment was replicated three times, and data were analyzed using Microsoft Office Excel 2019. Correlation analysis and principal component analysis (PCA) were performed using IBM SPSS Statistics (Version 22.0; IBM SPSS Inc., USA) based on the method described by Jin [61]. Multiple comparisons were made using Duncan’s test at a significance level of *p* < 0.05 [27]. Graphs were generated using Prism 9.0 statistical software (https://www.graphpad-prism.cn, accessed on 2 February 2026). Tbtools (v2.091, https://bio.tools/tbtools, accessed on 29 January 2026) and Chiplot online (https://www.chiplot.online/, accessed on 30 January 2026) were used for cluster analysis.

The greatest impact of combination stress treatments on plants was determined using the membership function average (MV) evaluation method in fuzzy mathematics [54]. The fuzzy membership function is expressed as follows. The different stress treatment concentrations were assessed using the final membership function (MV). The higher the MV, the better the treatment concentration.

$$V_{\mathrm{ij}a}=\left(X_{\mathrm{ij}}-X_{j\min}\right)/\left(X_{j\max}-X_{j\min}\right)$$

$$V_{\mathrm{ij}b}=1-\frac{\left(X_{\mathrm{ij}}-X_{j\min}\right)}{\left(X_{j\max}-X_{j\min}\right)}$$

$$MV_{i}=\frac{1}{n}\sum_{j=1}^{n}V_{\mathrm{ij}}$$

where *V_ij_*_a_ and *V_ij_*_b_ are the membership function values of index j, and *V_ij_*_a_ and *V_ij_*_b_ represent the positive and negative correlations with the treatment, respectively. *X_ij_* is the measured mean value of the index for different stress concentrations, and *X_j_*_max_ and *X_j_*_min_ are the maximum and minimum values of index *j*, respectively. *MV_i_* is the mean value of the affiliation function of all indicators under different stress treatments.

### 4.7. Statistical Analyses for Physiological and Molecular Indicators

All statistical methods described in this subsection were applied to the physiological parameters and gene expression data determined in this study. All values between the control and treatment groups are given as means ± SD (*n* = 3). Significant differences were determined using one-way ANOVA using the IBM SPSS Statistics (Version 22.0; IBM SPSS Inc., USA), followed by Tukey’s HSD multiple comparison test, and are indicated with asterisks as follows: * *p* < 0.05; ** *p* < 0.01; ns, no significant difference.

## 5. Conclusions

In this study, we aimed to investigate the physiological and molecular responses of wheat to single and combined stress treatments to explore the mechanisms of wheat seedlings’ response to different stresses. In this study, drought stress simulated with PEG exerted stronger inhibitory effects on seed germination, seedling growth, and physiological indexes than salt stress simulated by NaCl, and these impacts were further aggravated under combined stress conditions, with the 20% DHS treatment causing the most severe growth reduction. Wheat copes with oxidative and osmotic damage under stress conditions through enhancing antioxidant enzyme activities, accumulating osmoprotectants, and regulating the expression of stress-responsive genes. Furthermore, through our analysis, we found that combined stresses caused more severe inhibitory effects on wheat than individual stresses, with drought–heat stress causing the strongest suppression. Notably, the drought–heat and salt–heat combined stresses induced distinct physiological and molecular adaptive mechanisms. These findings offer an understanding of wheat responses to multiple abiotic stresses.

## Figures and Tables

**Table 1:** Germination performance of wheat seeds treated with single and combined stress.

Treatment		Germination Energy (GE)/%	Germination Percentage (GP)/%	Germination Index (GI)
Normal	CK	85.19 ± 1.70	92.22 ± 1.92	66.99 ± 0.97
	DS-10%	77.47 ± 1.28 **	84.44 ± 1.92 **	53.79 ± 1.08 **
	DS-15%	78.15 ± 1.70 **	83.33 ± 3.33 *	50.53 ± 2.36 **
	DS-20%	72.22 ± 1.92 **	82.22 ± 1.92 **	51.75 ± 3.05 **
	SS-0.15%	84.07 ± 1.28	88.89 ± 3.85	69.89 ± 0.77 *
	SS-0.45%	85.56 ± 1.70	87.78 ± 3.85	68.32 ± 0.99
	SS-0.75%	84.81 ± 1.92	85.56 ± 1.92 *	68.94 ± 1.45
Heat	HS	75.56 ± 1.92	80.00 ± 3.33	50.61 ± 3.14
	DHS-10%	66.67 ± 3.33 *	65.56 ± 1.92 **	44.73 ± 2.34
	DHS-15%	65.56 ± 1.92 **	62.22 ± 3.85 **	41.78 ± 1.67 *
	DHS-20%	56.67 ± 3.33 **	55.56 ± 1.92 **	40.56 ± 0.95 **
	SHS-0.15%	72.22 ± 3.85	72.22 ± 1.92 *	52.35 ± 3.11
	SHS-0.45%	67.78 ± 1.92 **	66.67 ± 3.33 **	47.72 ± 1.86
	SHS-0.75%	56.67 ± 3.33 **	56.67 ± 3.33 **	41.91 ± 2.06 *

Note: All data are presented as mean ± SD (*n* = 3). Significant differences were determined using one-way ANOVA followed by Tukey’s HSD multiple comparison test. * *p* < 0.05, ** *p* < 0.01 compared with the corresponding control (CK for the normal temperature group, HS for the high temperature group).

**Table 2:** The length of wheat leaf or roots treated with single and combined stress.

Treatment		Leaf Length		Root Length	
		3d	7d	3d	7d
Normal	CK	11.67 ± 0.07	12.79 ± 0.16	15.34 ± 0.29	16.42 ± 0.26
	DS-10%	11.16 ± 0.12 **	11.23 ± 0.15 **	13.87 ± 0.28 **	12.80 ± 0.27 **
	DS-15%	9.04 ± 0.08 **	10.47 ± 0.31 **	13.47 ± 0.13 **	12.43 ± 0.21 **
	DS-20%	9.07 ± 0.24 **	9.89 ± 0.34 **	12.57 ± 0.24 **	12.88 ± 0.12 **
	SS-0.15%	11.32 ± 0.43	11.60 ± 0.27 **	12.90 ± 0.20 **	12.35 ± 0.09 **
	SS-0.45%	10.69 ± 0.77	10.85 ± 0.18 **	11.07 ± 0.35 **	11.11 ± 0.07 **
	SS-0.75%	9.71 ± 0.38 **	11.41 ± 0.16 **	11.01 ± 0.20 **	11.05 ± 0.20 **
Heat	HS	11.88 ± 0.31	10.51 ± 0.26	13.60 ± 0.23	13.56 ± 0.05
	DHS-10%	11.62 ± 0.08	10.45 ± 0.42	12.33 ± 0.14 **	12.13 ± 0.09 **
	DHS-15%	9.59 ± 0.13 **	8.98 ± 0.15 **	11.22 ± 0.19 **	10.35 ± 0.21 **
	DHS-20%	9.53 ± 0.23 **	8.51 ± 0.18 **	10.95 ± 0.15 **	10.62 ± 0.30 **
	SHS-0.15%	9.96 ± 0.45 **	10.13 ± 0.20	11.28 ± 0.14 **	10.98 ± 0.15 **
	SHS-0.45%	8.66 ± 0.21 **	8.43 ± 0.35 **	10.55 ± 0.17 **	10.20 ± 0.14 **
	SHS-0.75%	8.71 ± 0.16 **	8.79 ± 0.27 **	10.59 ± 0.09 **	10.39 ± 0.28 **

Note: All data are presented as mean ± SD (*n* = 3). Significant differences were determined using one-way ANOVA followed by Tukey’s HSD multiple comparison test. ** *p* < 0.01 compared with the corresponding control (CK for the normal temperature group, HS for the high temperature group).

## Data Availability

Data are contained within this article and the Supplementary Materials.

## Supplementary Materials

The following supporting information can be downloaded at https://www.mdpi.com/article/10.3390/ijms27115126/s1.
