# Supplementary figures and images for: Physiological, Morphological, and Molecular Evaluation of Wheat Under Single (Drought, Salt, Heat) and Combined (Drought–Heat, Salt–Heat) Stress
Source: Int J Mol Sci. 2026 Jun 5;27(11):5126. doi: 10.3390/ijms27115126 (PMC13258611; doi:10.3390/ijms27115126)

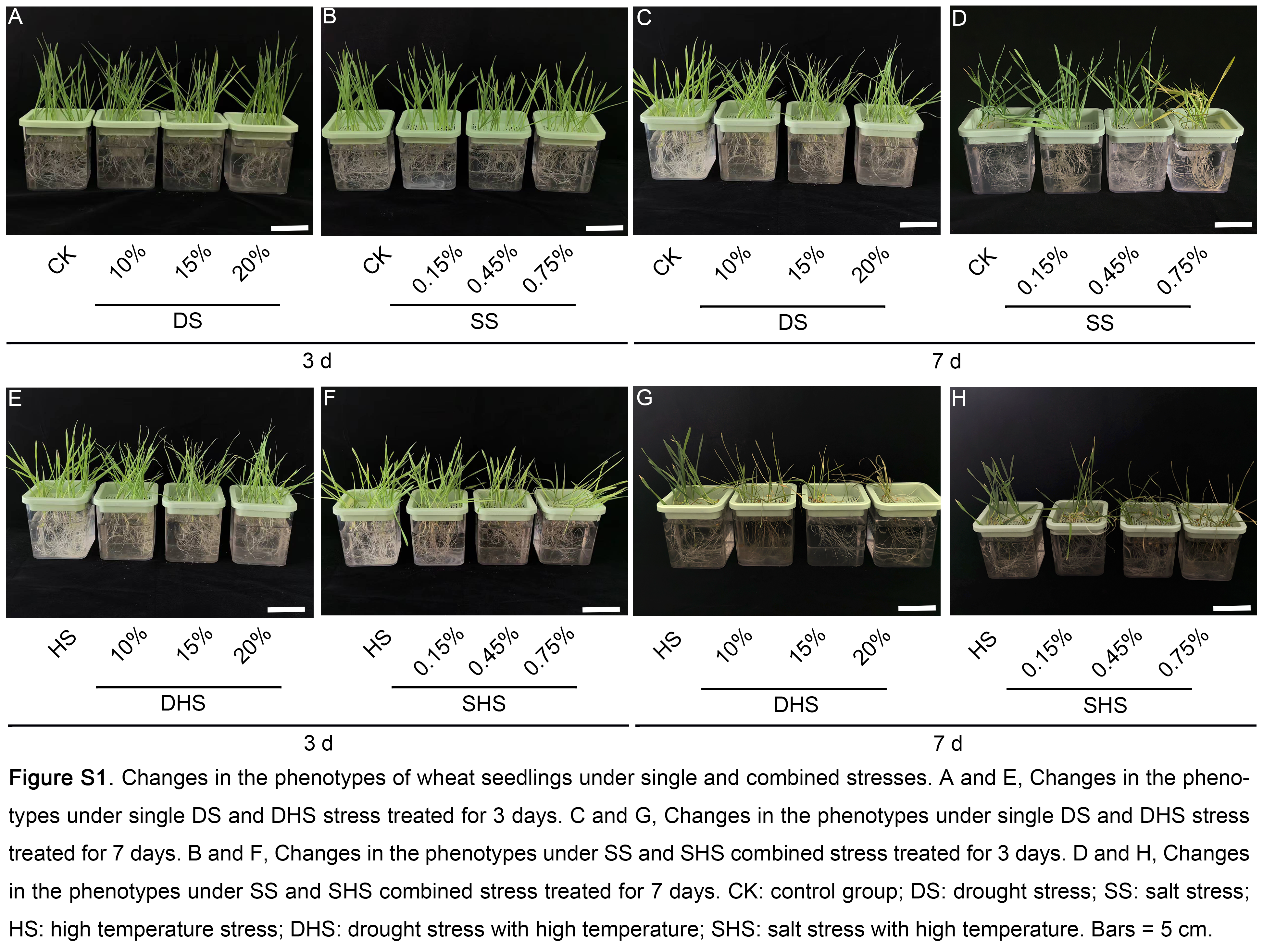

Supplement: Supplementary file 1 [file ijms-27-05126-s001.zip › Figure S1.tif]

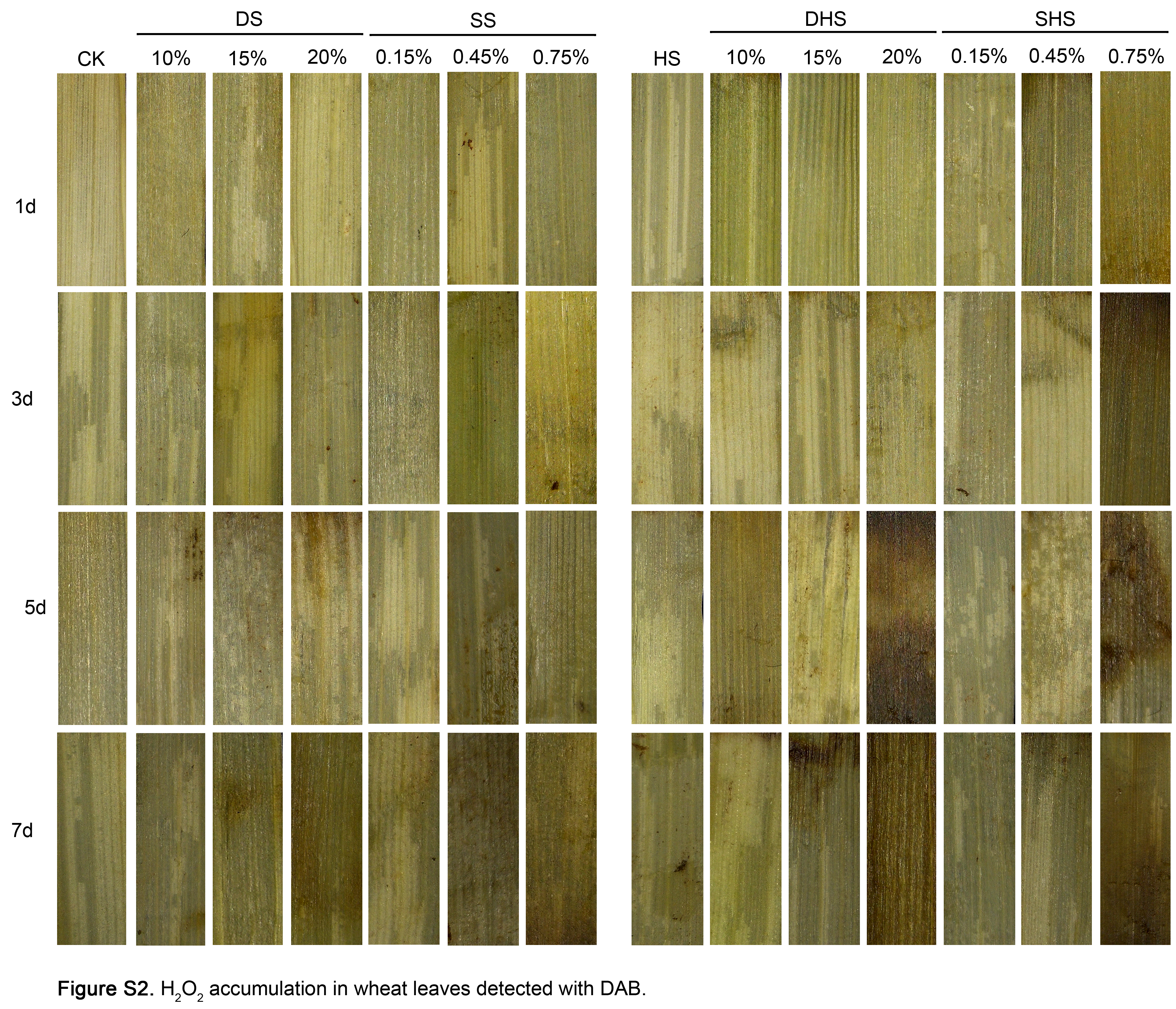

Supplement: Supplementary file 1 [file ijms-27-05126-s001.zip › Figure S2.tif]

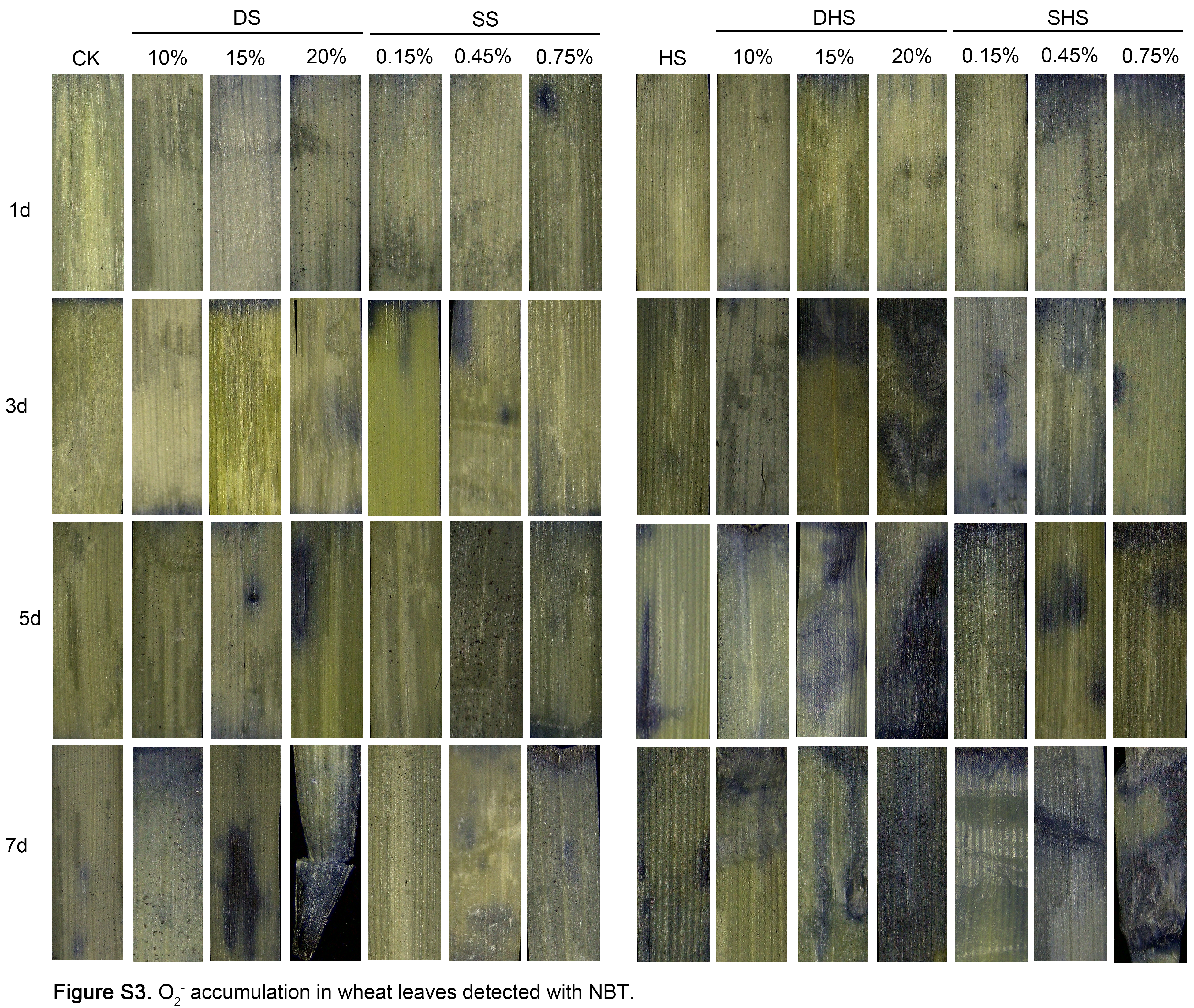

Supplement: Supplementary file 1 [file ijms-27-05126-s001.zip › Figure S3.tif]
